# Understanding dynamics using sensitivity analysis: caveat and solution

**DOI:** 10.1186/1752-0509-5-41

**Published:** 2011-03-15

**Authors:** Thanneer M Perumal, Rudiyanto Gunawan

**Affiliations:** 1 Department of Chemical and Biomolecular Engineering, National University of Singapore, Singapore; 2 Institute for Chemical and Bioengineering, ETH Zurich, Switzerland

## Abstract

**Background:**

Parametric sensitivity analysis (PSA) has become one of the most commonly used tools in computational systems biology, in which the sensitivity coefficients are used to study the parametric dependence of biological models. As many of these models describe dynamical behaviour of biological systems, the PSA has subsequently been used to elucidate important cellular processes that regulate this dynamics. However, in this paper, we show that the PSA coefficients are not suitable in inferring the mechanisms by which dynamical behaviour arises and in fact it can even lead to incorrect conclusions.

**Results:**

A careful interpretation of parametric perturbations used in the PSA is presented here to explain the issue of using this analysis in inferring dynamics. In short, the PSA coefficients quantify the integrated change in the system behaviour due to persistent parametric perturbations, and thus the dynamical information of when a parameter perturbation matters is lost. To get around this issue, we present a new sensitivity analysis based on impulse perturbations on system parameters, which is named impulse parametric sensitivity analysis (iPSA). The inability of PSA and the efficacy of iPSA in revealing mechanistic information of a dynamical system are illustrated using two examples involving switch activation.

**Conclusions:**

The interpretation of the PSA coefficients of dynamical systems should take into account the persistent nature of parametric perturbations involved in the derivation of this analysis. The application of PSA to identify the controlling mechanism of dynamical behaviour can be misleading. By using impulse perturbations, introduced at different times, the iPSA provides the necessary information to understand how dynamics is achieved, i.e. which parameters are essential and when they become important.

## Background

Parametric sensitivity analysis (PSA) has become a must have tool in the computational systems biologists' arsenal. In most applications of this analysis, one computes sensitivity coefficients or metrics, which generally reflect the ratios between the change in a biological model output and the perturbation on system parameters that cause this change. Depending on the magnitude of the perturbations, sensitivity analyses can be classified into local (infinitesimal perturbation) and global (finite perturbation). Regardless of these classes, the interpretation of the sensitivity metrics is intuitive; parameters with large sensitivity magnitude are deemed to be important and hence considered to be the controlling factors in the system functional regulation. Consequently, one of the common uses of PSA in systems biology is to infer the importance of cellular processes or pathways and to provide mechanistic explanations for biological behaviour [1-5].

On a separate note, dynamics is a prominent feature of many important biological processes (e.g., oscillations in cell cycle and circadian rhythm [6,7], switching behaviour in programmed cell death [8], and adaptation in chemotaxis [9]). Cellular homeostatic regulation, despite the name, relies on an active dynamical response, in which orchestrated events take place in response to internal and external stimuli. Thus, understanding cellular dynamics has become a prime concern in systems biology, in which mathematical modelling coupled with quantitative analysis have been used to gain insights on the mechanisms that give rise to and control the dynamic behaviour[1-5]. These insights can provide the molecular targets for altering system dynamic behaviour, such as in finding treatment for diseases or in (re)engineering of cellular systems.

While there are many choices of mathematical frameworks for dynamic modelling, ordinary differential equations (ODEs) are the most commonly used modelling paradigm in systems biology and have been used to describe a wide range of biological systems. In addition, ODEs are amenable to many standard quantitative and theoretical analyses, including sensitivity analysis and bifurcation analysis, for which many off-the-shelf software packages exist that provide an integrated and user-friendly computational platform for model simulations and analyses (e.g., MATLAB [10] and XPPAUT [11]). The PSA of ODE models can be readily done using software packages such as SimBiology toolbox of MATLAB [12], PottersWheel [13], Gepasi [14], Copasi [15], JDesigner/Jarnac [16], JSim [17], BioSens [18], SBML-SAT [19], and SensSB [20]. These and other software for sensitivity analysis have been summarized in the review articles by Alves. *et al. *[21] and Klipp. *et al. *[22].

Sensitivity analysis of ODE models is well established in the science and engineering literature [23-32]. In systems biology, PSA has found wide applications, such as for model calibration and identifiability, model validation and reduction, identification of bottlenecking processes, elucidation of mechanisms of complex cellular behaviour, and investigation of cellular robustness [30,33]. A few notable examples of PSA applications in dynamic biological models include programmed cell death [34-39], budding yeast cell cycle control [6], IL-6 signalling pathway [1], circadian rhythm models [7,40,41], and coupled MAPK and PI3K signal transduction pathway [42]. In many applications, PSA is used to generate parameter ranking based on the magnitude of sensitivity coefficients, either taken at a specific time or using consolidated sensitivity metrics, such as time-integral or average or norm of sensitivity coefficients [34,43,44]. The parameter ranking is subsequently used to conclude about the mechanism or property (such as robustness) of the biological system behaviour [1-5].

In this article, we show that the dynamical aspects of cellular functional regulation cannot be inferred from the sensitivity coefficients of PSA, neither directly nor as consolidated sensitivity metrics. More importantly, the corresponding parameter rankings from PSA can give erroneous inference about the controlling mechanisms. Briefly, the reason stems from the fact that in PSA, perturbations are introduced on system parameters, which are time-invariant or static. In other words, these parametric perturbations are persistent and their effects on the system behaviour are integrated over time. Therefore, while PSA can indicate which parameter perturbations are important, it does not point to when these perturbations matter. This problem is illustrated using local PSA of two examples: a synthetic network and a model of programmed cell death [37]. Although the illustration here was done using local sensitivity analysis, the same issue generally applies to global PSA.

To overcome this issue, a new parametric sensitivity analysis is developed in this work. This analysis differs from the classical PSA in the manner of which perturbations are introduced on model parameters, specifically using impulses, and thus is named impulse parametric sensitivity analysis (iPSA). By analyzing the consequence of impulse parameter perturbations introduced at different times, the iPSA provides time-varying, mechanistic explanation on how system dynamics is carried out. The new insights from the iPSA are demonstrated using the same two examples mentioned above.

## Results and discussion

### Simple network model

**Figure 1:**
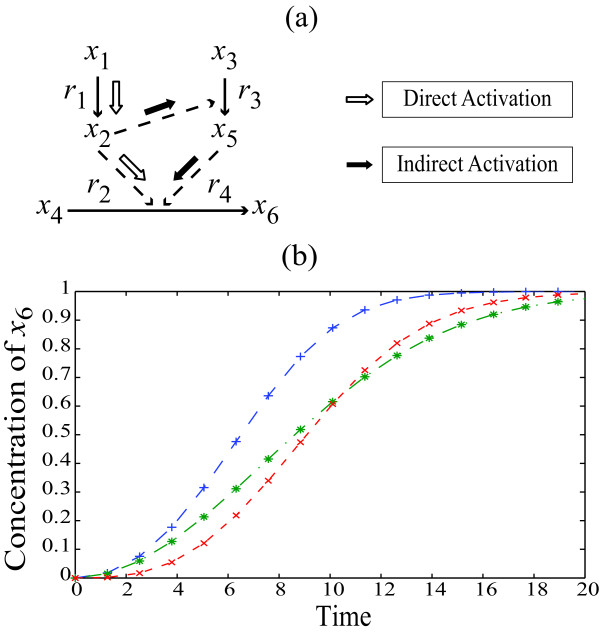
**A simple network model**. (a) A simple network with 6 states and 4 reactions. Straight arrows connect substrate to product and the dotted arrows indicate enzymatic activity. Details of model equations and parameter values are given in Table S1 (see Additional file 1: Supplementary Table S1). (b) Activation of *x_6 _*under a constant stimulus of *x*_1 _= 1: complete network (+), indirect pathway knock-out (*) and direct pathway knockout (x).

To illustrate the shortcoming of local PSA in analyzing system dynamics, consider a simple six state model involving three reactions with Michealis-Menten (MM) kinetics, as shown in Figure 1(a) (model parameters, rate kinetics and initial concentrations are given in Additional File 1: Supplementary Table S1). In this network, the activation of *x*_6 _followed a switch-like dynamics in response to the stimulus *x*_1_, as illustrated in Figure 1(b) (nominal). The model describes two pathways that contribute to *x*_6 _activation: a direct *x*_2 _pathway and an indirect *x*_2_, *x*_3_, and *x*_5 _pathway.

In this example, *in silico *knock-out experiments were performed by removing each pathway individually in order to assess the dominance of one pathway over the other in *x*_6 _activation. Both full network and knock-out (KO) simulations were performed under a stimulus of *x*_1_(*t*_0 _= 0) = 1. As illustrated in Figure 1(b), while the initial *x*_6 _activation in the indirect pathway KO remained the same as that of the original model, the switch-like activation was much less pronounced. On the other hand, the original switching behaviour was preserved in the direct pathway KO, but the switching time was delayed due to a slower initial activation. Taken together, these KO simulations suggested that the *x*_6 _activation is mainly accomplished through the indirect pathway, while the direct pathway contributes mainly to the initial *x*_6 _activation.

### Parametric sensitivity analysis for dynamical systems: A caveat

Local parametric sensitivity analysis was also used to study the pathway dominance in this simple network. Mathematically, the parametric sensitivity coefficient is given as(1)

where *x_i _*is the *i*-th state in an ODE model and *p_j _*is the *j*-th kinetic parameter of an ODE model (for a detailed description of sensitivity coefficient derivation, see Methods). These sensitivity coefficients describe the change in system output (state trajectory) at time *t *with respect to (an infinitesimal) perturbation on the system parameter values at time *τ*. Here, the PSA was performed for the same stimulus *x*_1_(0) = 1 with *τ *= 0 and the sensitivity coefficients were computed for the time range of 0-15 time units. The term *local *sensitivity analysis refers to the fact that the results will depend on the nominal parameter values around which the derivatives in Eq. (1) are calculated.

**Figure 2:**
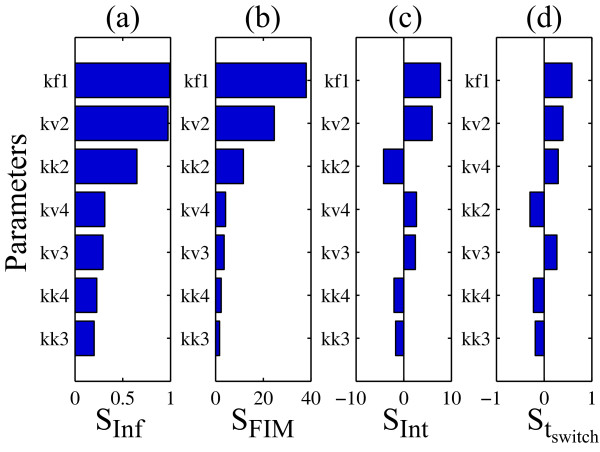
**Local parametric sensitivity analysis of *x*_6 _activation under *x*_1 _stimulus**. The bar graphs show the consolidated sensitivity metrics of *x_6 _*with respect to model parameters based on (a) infinite norm, (b) Fisher Information Matrix (FIM) and (c) time integrated sensitivity coefficients; and (d) the sensitivity magnitudes at switching time (*t *= 7.12 time units). The parameter numbers refer to the reactions as shown in Figure 1, where the subscripts *f *denotes the forward rate constant and *k *and *v *denotes the rate constants of Michealis-Menten kinetics.

The sensitivities of *x*_6 _with respect to all model parameters are ranked in Figure 2 using consolidated sensitivity metrics, i.e. infinite norm [44] (Figure 2(a)), FIM [43] (Figure 2(b)), and time-integral [34] (Figure 2(c)), and using sensitivity magnitudes at switching time (*t *= 7.12 time units; Figure 2(d)). The conclusion from these rankings was the same: (1) the largest sensitivity was associated with the kinetics of *x*_1 _conversion to *x*_2 _and (2) the direct pathway (*r*_2_) parameters have larger sensitivities than those from the indirect pathway (*r*_4_), suggesting larger influence of the direct pathway on the *x*_6 _activation. Hence, the conclusion from the PSA is in direct contradiction with the findings from *in silico *KO experiments.

**Figure 3:**
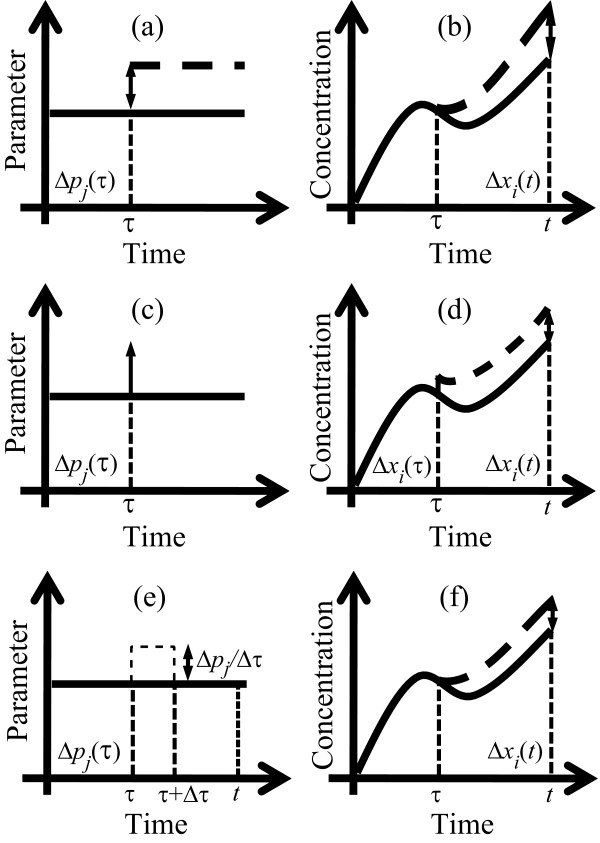
**Parametric perturbations in sensitivity analysis**. An illustration of parametric perturbation and its effect on system dynamics in (a-b) PSA, (c-d) iPSA and (e-f) pulse-approximation of iPSA. Solid lines represent the nominal and the dashed lines show the perturbed trajectory, respectively. Figures are not drawn to scale.

This discrepancy can be explained by taking a closer look at the way parametric sensitivity coefficients in eqn. (1) are calculated:(2)

where *τ *= *t*_0 _is the usual perturbation time,  is the time derivative of sensitivity coefficient  (see Methods for detail) and *H*(*t*) is the Heaviside step function. In this case, since model parameters are static or time-invariant, the parametric perturbations in the PSA consist of step changes in the parameter values, as depicted in Figure 3(a). Hence, the sensitivity coefficients at time *t *represent an integrated or accumulated change in the states from *τ *to *t *due to a persistent parameter change started at time *τ *(see Figure 3(b)). Indeed, substituting the full equation of  (see Methods) in eqn. (2) gives(3)

Here, one sees two terms in the integrand that contribute to the sensitivity coefficients at time *t*: (1) the first is related to the (integrated) sensitivities that are carried over from the initial perturbation time *τ *and (2) the second accounts for the instantaneous rate changes due to the parametric perturbations that still persist at time  Thus, in the PSA, a large sensitivity magnitude of *S_*i*,*j*_*(*t*,*τ*) indicates the importance of the *j*-th parameter in time window of *τ *and *t*, during which the perturbation is applied to the system. Hence, the use of these coefficients to infer the dynamical importance of parameters is inappropriate and can even be misleading.

For the reason above, the PSA of the simple network model gave an incorrect conclusion regarding direct versus indirect pathway activating *x*_6_. As seen in the *in silico *KO experiments, the direct pathway regulates the initial activation of *x*_6_, while the actual switching is carried out by the indirect pathway. In the PSA of this model (*τ *= 0), the early importance of the direct pathway and also the reaction *r*_1 _persisted beyond the initial times in the sensitivity coefficients due to the aforementioned integrated effect. In this case, the importance of the indirect pathway was not apparent from the parameter sensitivity rankings in the background of large (integrated) sensitivities with respect to *r*_1 _and the direct pathway. Correspondingly, the use of any time-consolidated sensitivity metrics will only worsen this problem.

### Impulse parametric sensitivity analysis (iPSA)

As the problem with local PSA mentioned above is rooted from the persistent parameter perturbations, which is also done in global PSA [45], a new sensitivity analysis is formulated here that introduces impulse perturbations to model parameters as shown in Figure 3(c-d). The corresponding impulse sensitivity coefficients *iS_*i*,*j*_*(*t*,*τ*) reflect the change in the *i*-th state *x_i _*at time *t *due to an impulse perturbation on the *j*-th parameter *p_j _*at time *τ *(see Methods for the derivation and definition). Since impulse perturbations on parameters cause an immediate state changes at the perturbation time (see Methods), the inference of dynamical parametric importance can be obtained from impulse sensitivities by varying the time of perturbation. However, as with the local PSA, impulse perturbations are also *local *in nature and thus the impulse sensitivities will depend on the nominal parameter values. The global equivalent of iPSA can be formulated using pulse perturbations and is currently under development. Finally, from time-varying impulse perturbations, the iPSA can give answer to the following questions about state dynamics: which are the important parameters and when do they become important?

**Figure 4:**
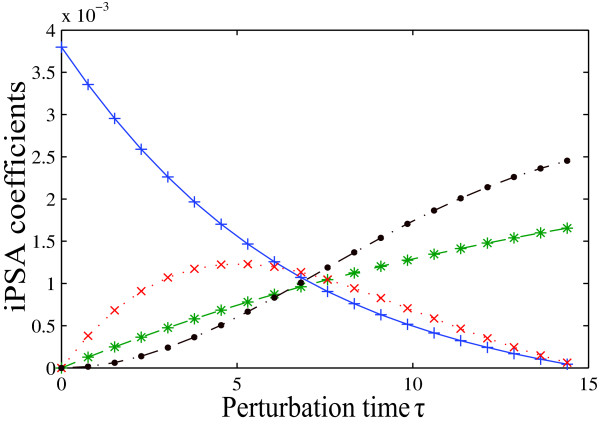
**Impulse parametric sensitivity analysis of *x*_6 _activation under x_1 _stimulus**. iPSA coefficients of *x*_6 _with respect to impulse parametric perturbations in kf1(+), kv2(*), kv3(x) and kv4 (•). The parameter definitions and values are reported in Additional file 1: Supplementary Table S1.

The iPSA was also applied to the simple network model under the same stimulus and for the same time range as above. Since the impulse perturbations are delivered at different times, the impulse sensitivity *iS_*i*,*j*_*(*t*,*τ*) has two-time dependence, with respect to the time of perturbation (*τ*) and the time of observation (*t*). Figure 4 shows the iPSA sensitivity coefficients of *x*_6 _at the end of simulation time (*t *= 15 time units), with respect to the four most important parameters at different perturbation times (for complete iPSA sensitivities, see Additional File 1: Supplementary Figure S1). In agreement with the KO simulations and in contrast to the findings from PSA, the impulse sensitivities gave support to the dominance of the indirect pathway. Specifically, the results showed that *x*_6 _activation: (1) is initiated by *r*_1 _(high initial sensitivity to parameter kf1); (2) is accomplished mainly by the direct pathway prior to the switching time (sensitivity to *r_2 _*is higher than to *r*_4 _during these times); and (3) is subsequently carried by the indirect pathway (highest sensitivity to *r*_4 _during switching times). A higher resolution analysis using a heat map of the complete iPSA coefficients with *t *and *τ *between 0 and 15 time units gave the same conclusion (see Additional file 1: Supplementary Figure S2).

### Fas-induced cell death model of human Jurkat T-cells

**Figure 5:**
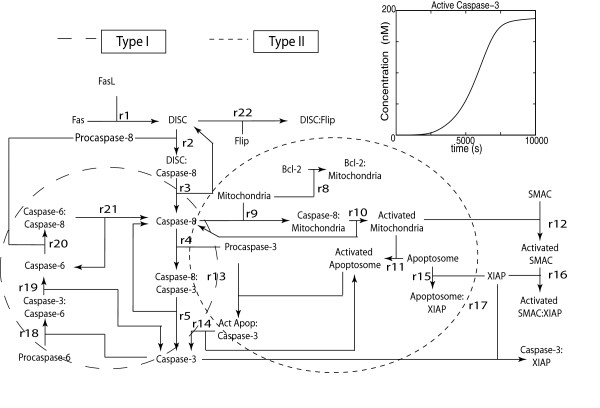
**Fas-induced apoptotic cell death model of human Jurkat T-cell lines **[37]. Detailed model equations and parameter values are given in Additional file 1: Supplementary Table S2. The activation of cell death effector, caspase-3 follows a switch like response to a constant FasL stimulus of 2 nM (see inset).

In the second case study, we consider a more complex biological network taken from the modelling of programmed cell death in Jurkat cancer T-cells (see Figure 5). The model equations and parameters were identified from experimental data [37] (see Additional file 1: Supplementary Table S2 for detailed reaction rates and parameters). In this network, the cell death is decided by the cleaving of procaspase-3 into caspase-3 [46], in response to FasL stimulus. The model simulation showed that caspase-3 is switched ON at around *t *= 6000 seconds (see Figure 5, inset) and like the simple network above, there exist two activating pathways: the direct caspase-8 (type-I) and the indirect mitochondria-dependent pathway (type-II). As in the previous case study, the classical PSA and iPSA were applied to this network to elucidate the pathway dominance.

**Figure 6:**
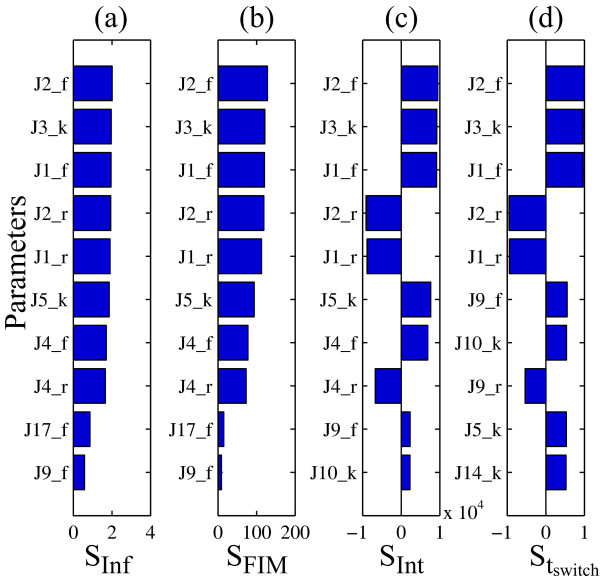
**Local parametric sensitivity analysis of the programmed cell death model**. (a-c) The bar graphs represent the ten largest sensitivity metrics of caspase-3 based on infinite norm, Fisher Information Matrix (FIM), time integrated sensitivity coefficients, and sensitivity magnitudes at the switching time (*t *= 6060s). The complete parameter ranking is given in Additional file 1: Supplementary Figure S3. The parameter numbers refer to the reactions shown in Figure 5, where the subscripts *f *and *r *denote forward and backward rate constants for reversible reactions and *k *denotes the rate constants for irreversible reactions.

**Figure 7:**
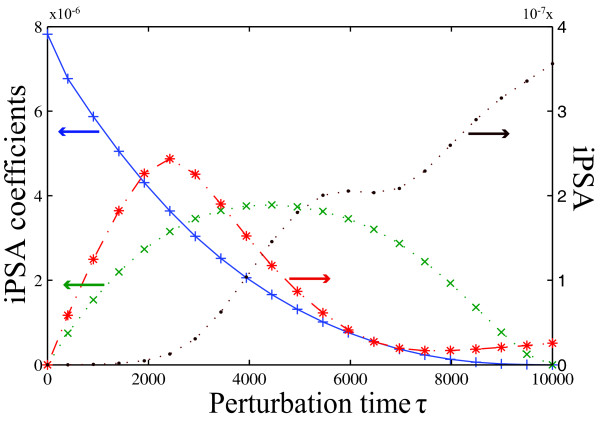
**Impulse parametric sensitivity analysis of the programmed cell death model**. The iPSA sensitivity of caspase-3 with respect to impulse parametric perturbations in J1_f(+), J5_k(*), J9_f(x) and J14_k(•). The complete iPSA sensitivities are given in Additional file 1: Supplementary Figures S4 and S5. The parameter numbers refer to the reactions shown in Figure 6, where the subscripts *f *and *r *denote forward and backward rate constants for reversible reactions and *k *denotes the rate constants for irreversible reactions.

The classical and iPSA analyses were calculated under a constant FasL stimulation (FasL = 2 nM) over the time range of 10,000 seconds. The rankings of the important parameters that control caspase-3 according to the PSA are shown in Figure 6, using consolidated metrics: infinite norm [44](Figure 6(a)), FIM [43] (Figure 6(b)), and time integral [34](Figure 6(c)), and using the sensitivity magnitudes at switching time (Figure 6(d)) (see Additional file 1: Supplementary Figure S3 for detailed sensitivity rankings). From these rankings, one could not obtain any definitive conclusion regarding the dominance of one pathway over the other. On the contrary, the iPSA sensitivity of caspase-3 in Figure 7 clearly supported a type-II dependent caspase-3 switching with an early type-I dependent activation, in agreement with two previous analyses of this model using the Green's function matrix [47] and model reduction [48]. In Figure 7, the high caspase-3 sensitivities with respect to impulse perturbations to upstream reactions were expected during the initial times, as these served as the early cell death signal response. The cleaving of procaspase-3 was carried out by caspase-8 directly (*r*_5 _of type-I) before *t *= 4000 seconds, after which the mitochondrial pathway (*r*_14 _of type-II) became the main route of activating caspase-3 (see Additional file 1: Supplementary Figures S4 and S5 for more detail).

**Figure 8:**
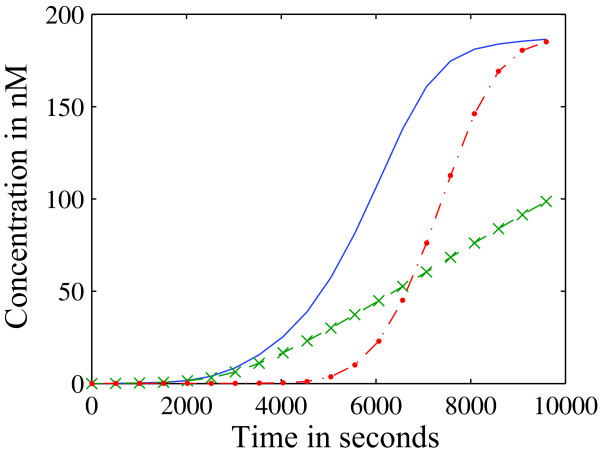
***In silico *knock-out of the programmed cell death network**. The activation of caspase-3 are simulated for nominal network (-) and reduced networks by knocking out type-I (•) and type-II pathway (x).

The discrepancy between the PSA and iPSA results can again be explained in the context of persistent versus impulse perturbations. As seen in the PSA parameter rankings in Figure 6 and following the insights offered by the iPSA in Figure 7, the effect of perturbing early response processes, including type-I pathway, was integrated over time in the PSA. For example, the highest ranked parameters in the PSA were associated with the first three reactions, *r*_1 _to *r*_3_. Such integration masked the dynamical importance of different parameters in this analysis. The conclusion from the iPSA is in agreement with the simulations of KOs of type-I and type-II pathways in Figure 8. While type-II knock-out could describe the caspase-3 activation at early times, it failed to capture the switching behaviour. On the other hand, the type-I knock-out was able to reproduce the switching of caspase-3, albeit with a short delay.

The two examples above illustrate the problem of using the classical PSA in identifying the controlling mechanisms of a dynamical system. Of course, this does not mean that the PSA of dynamical models is incorrect, but rather the interpretation of the sensitivity coefficients should be carefully managed. In particular, a large sensitivity magnitude with respect to a parameter suggests the importance of this parameter in the time period between the perturbation time *τ *and the state observation time *t*. In contrast, the iPSA is developed with dynamics in mind, where the impact of a single perturbation on the system is realized only at the perturbation time and subsequently they are delivered at varying perturbation times. By doing so, the iPSA coefficients can elucidate the way system dynamics **x**(*t*) is achieved, by indicating which and when parameters or processes are essential. Because of the persistent nature of perturbations used in the PSA, it is still not possible to reproduce the conclusions of the iPSA by varying the time of perturbation (see Additional file 1: Supplementary Figures S6 and S7).

## Conclusions

While classical parametric sensitivity analysis provides a powerful tool to understand the parametric dependence of biological behaviour, its suitability in inferring mechanisms of dynamic behaviour has not been properly addressed. The two case studies here illustrated the caveat of using local PSA for such purpose. The issue mainly arose from the information needed to do this inference, where one needs to know not only which parameters are critical, but also when they matter. However, the persistent parametric perturbations in standard PSA are incapable of providing this information as the sensitivity coefficients represent an integrated effect. A new sensitivity analysis, called impulse parametric sensitivity analysis (iPSA), was developed with dynamical systems in mind. In particular, the iPSA makes use of local impulse perturbations introduced at different times to produce the necessary information for understanding dynamics. The application of iPSA to the case studies was able to correctly pinpoint the mechanisms responsible for dynamical system behaviour, while local PSA failed in these cases. Since the discrepancy between PSA and iPSA arises from a fundamental difference in the manner of which parametric perturbations are realized (i.e. persistent vs. impulse), the same caveat and solution can be generalized to the global PSA, in which the perturbations are no longer infinitesimal.

## Methods

### Mathematical Models

Ordinary differential equation models of dynamic systems can generally be written as:(4)

In biological models, the state **x **∈ ℝ^*n *^is typically the concentration vector of biomolecular species, such as mRNAs and proteins, while the function *f *is the constitutive, often nonlinear, rate equation. The right hand side of the ODE captures the generation and consumption of biomolecules due to a variety of processes in the cell (e.g. transcription, translation, phosphorylation and dephosphorylation, *etc*), the rates of which depend on a set of kinetic parameters that are consolidated in the vector . Since the initial conditions **x**_0 _can be treated in the same way as model parameters, the aggregate vector **p **∈ ℝ^*m*+*n *^is used here to denote the combined parameters and initial conditions, i.e. .

### Local parametric sensitivity analysis (PSA)

The effect of parameter perturbations can be written in a Taylor series expansion:(5)

where the partial derivatives ∂*x_i_*/∂*p_j_*'s are the first-order sensitivity coefficients, describing the linear change in the state *x_i _*at any time *t *with respect to an infinitesimal perturbation to the parameter *p_j_*. In general, the parametric perturbation can be introduced at any time *τ*(*t*_0 _ ≤ *τ *≤ *t*) [27], i.e.(6)

but in the PSA, the perturbation time *τ *is commonly taken to be the initial time *t*_0_. Hence, the argument *τ *is typically dropped out of eqn. (6) and the sensitivity coefficients only carry a single time dependence on the observation time *t *[1-3,5,30,44,49,50]. The higher order sensitivity coefficients in the Taylor series expansion are less commonly computed, and hence the focus of the current work is only on the first-order sensitivities. Because the magnitude of perturbations are infinitesimally small, the sensitivity coefficients will depend on the nominal or baseline parameter values, and thus the classical PSA is considered a local analysis.

The sensitivity coefficients of an ODE model can be computed by directly differentiating the model eqn. (4) with respect to parameters **p**, giving the differential equation for the sensitivity coefficients as:(7)

where ∂*f*/∂**x **is also known as the Jacobian matrix and **0**^*n*×*m *^and **I**^*n*×*n *^are *n × m *zero and *n × n *identity matrices, respectively. The parametric sensitivity coefficients in eqn. (7) need to be solved simultaneously with the ODE model in eqn. (4), which is called the direct method [28]. As the state and parameter values may span a large range of magnitudes, normalized sensitivity values are often used to compare among states and parameters and to generate parameter ranking, which is given by:(8)

In most systems biology applications of PSA, parameter rankings are generated from the sensitivity coefficients, either directly or using some consolidated sensitivity metrics. In this article, four sensitivity metrics are used to rank parameters based on the PSA results:(9)

where the indices *i *and *j *again denote the *i*-th state and *j*-th parameter, and *S*_inf_, *S*_FIM_, *S*_int _and  are the sensitivity metrics based on infinite norm [44], Fisher information matrix [43], time integral [34] and sensitivity magnitude at a particular time, respectively.

### Impulse parametric sensitivity analysis (iPSA)

The derivation of the iPSA coefficient follows the illustration in Figure 3(e). The sensitivity coefficients of iPSA are constructed by quantifying the ratio between the change in the state *x_i _*at time *t *and the causative pulse perturbation of size Δ*p_j _*/Δ*τ *for a duration of Δ*t*, which is applied to the parameter *p_j _*at time *τ*, in the limit Δ*p_j _*and Δ*τ *tending to zero. The first step of the derivation is to quantify the change in all states **x **at the end of the pulse perturbation, i.e. at time *τ *+ Δ*τ*, using the Taylor series expansion:(10)

In the next step, the change Δ**x**(*τ *+ Δ*τ*) is translated to the change in the state *x_i _*at time *t *using the Green's function matrix (GFM) **S**^*x *^(*t*,*τ *+ Δ*τ*) [47]. The (*i,j*)-th element of the GFM represents the sensitivity of the state *x_i _*at time *t *to alteration in the state *x_j _*at some previous time *τ*, i.e.(11)

Thus, the change Δ*x_i _*(*t*) due to the pulse perturbation is given by(12)

Subsequently, substitution of eqn. (10) in eqn. (12) gives(13)

Then, taking Taylor series expansion of the parametric sensitivities around the time *τ *and dividing both sides by Δ*p_j_*, one arrives with:(14)

Finally, taking the limit as Δ*p_j_*,Δ*τ *→ 0 such that the pulse perturbation becomes an impulse, the iPSA coefficient is obtained as:(15)

Note that at *t *= *τ*, the iPSA coefficient reduces to(16)

since **S**^*x*^(*τ*,*τ*) = **I**. In other words, the impulse parameter perturbation causes an immediate change in the state **x **at time *τ*. By rewriting eqn. (15) as:(17)

one can further see that the impact of this impulse perturbation takes effect only at the perturbation time *τ *and that the consequence on the state trajectory is equivalent to perturbing the states themselves, similar to the GFM analysis. Like in the PSA, the iPSA coefficients should be normalized for comparison and parameter ranking purposes, according to:(18)

## List of abbreviations

KO: Knock-Out; ODE: Ordinary Differential Equations; PSA: Parametric Sensitivity Analysis; iPSA: Impulse Parametric Sensitivity Analysis.

## Authors' contributions

TMP performed the experiments and wrote the manuscript. RG supervised the study and wrote the manuscript. All authors have read and approved the final manuscript.

## Supplementary Material

Additional file 1 **Supplementary Material**. Detailed results of iPSA and PSA, including model equations and parameters, of the simple network model and Fas-induced cell death model Click here for file
