# Supplementary material for: Understanding dynamics using sensitivity analysis: caveat and solution
Source: BMC Syst Biol. 2011 Mar 15;5:41. doi: 10.1186/1752-0509-5-41 (PMC3070647; doi:10.1186/1752-0509-5-41)
Supplement: Additional file 1 — Supplementary Material. Detailed results of iPSA and PSA, including model equations and parameters, of the simple network model and Fas-induced cell death model [file 1752-0509-5-41-S1.PDF]

Supplementary Information:

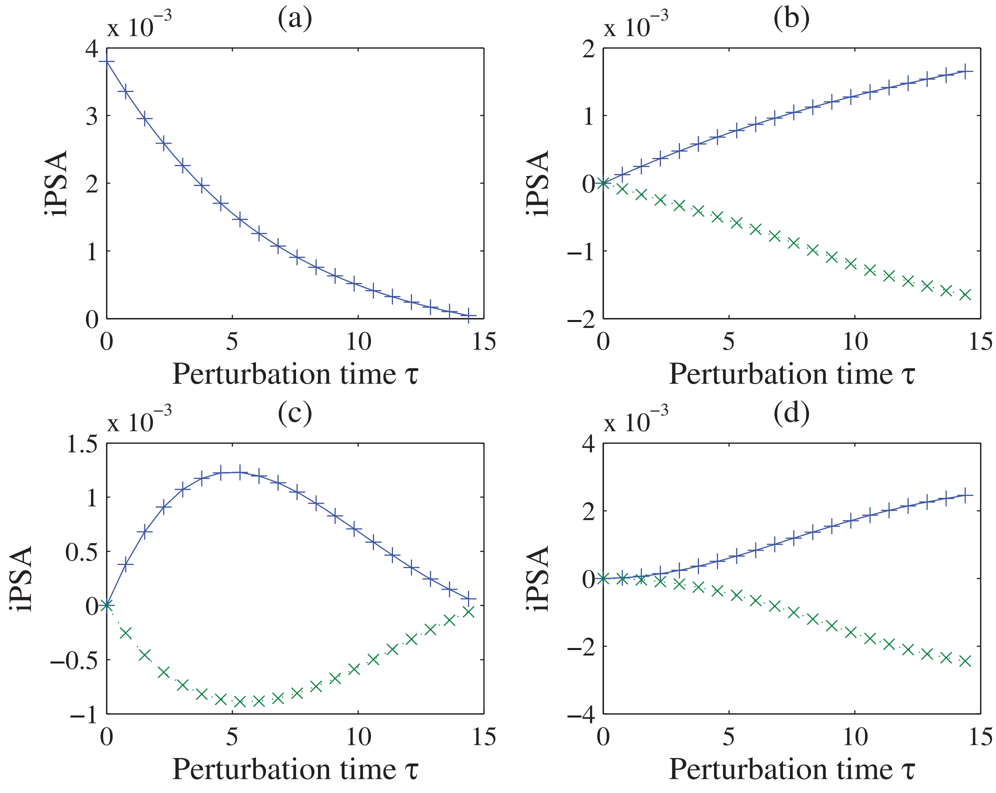

Supplementary Figure S1: Impulse Parametric Sensitivity Analysis (iPSA) of x_6 activation in response to x_1 stimulus at switching time (t = 7.12)

(a) kf1(+), (b) kv2(+) and kk2(x), (c) kv3(+) and kk3(x), (d) kv4(+) and kk4(x)

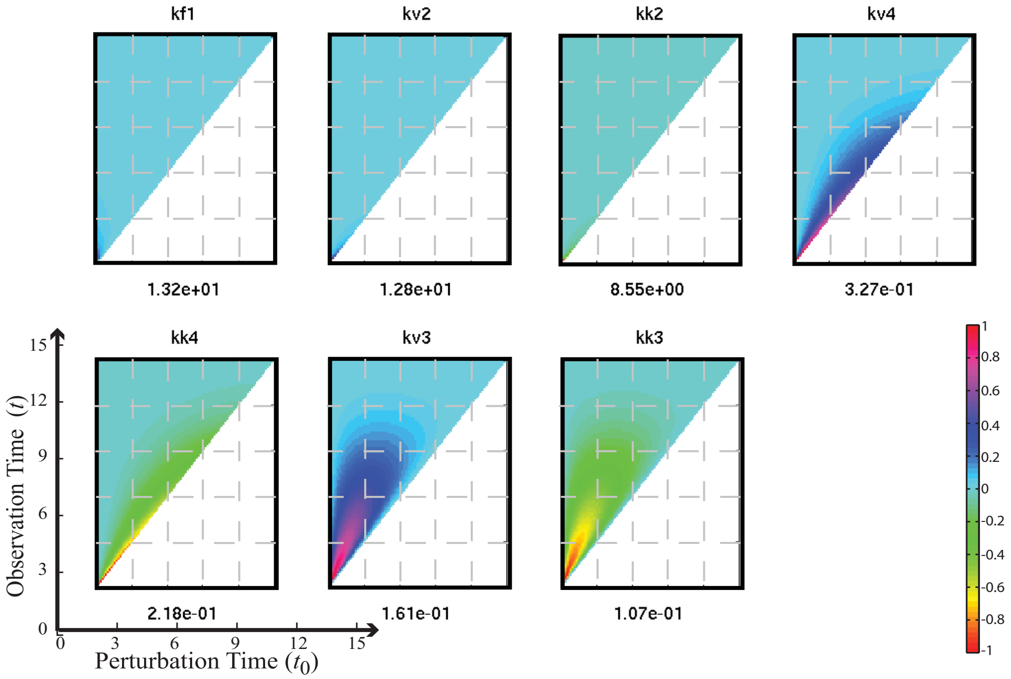

Supplementary Figure S2 - Impulse Parametric Sensitivity Analysis (iPSA) of x_6 activation in response to x_1 stimulus

Each heatmap illustrates the iPSA coefficient of active x_6 level with respect to perturbations on one parameter in the network, indicated in the title. The x-axis gives the time at which impulse perturbation is applied to the parameter, while the y-axis indicates the observation time of $x_6(t)$. For comparison purpose, each plot is scaled to have values between -1 and +1 by the scaling factor reported in the abscissa. The plots are arranged in the decreasing order of their scaling factors (left to right, top to bottom). The heatmap suggests that: (a) the early response to the stimulus depends on kf1, as indicated by the large sensitivity peak near the y-axis (early perturbation is important and the impact is delayed); (b) the early activation of x_6 depends on kv2 and kk2, as indicated by the sensitivity peaks along the diagonal (little or no delay in between perturbation and impact) during initial times; (c) the activation depends mainly on kv4 and kk4, as indicated by the peaks along the diagonal during the switching time; and finally (d) kv3 and kk3 control the intermediates, as the peaks lie in between the y-axis and the diagonal.

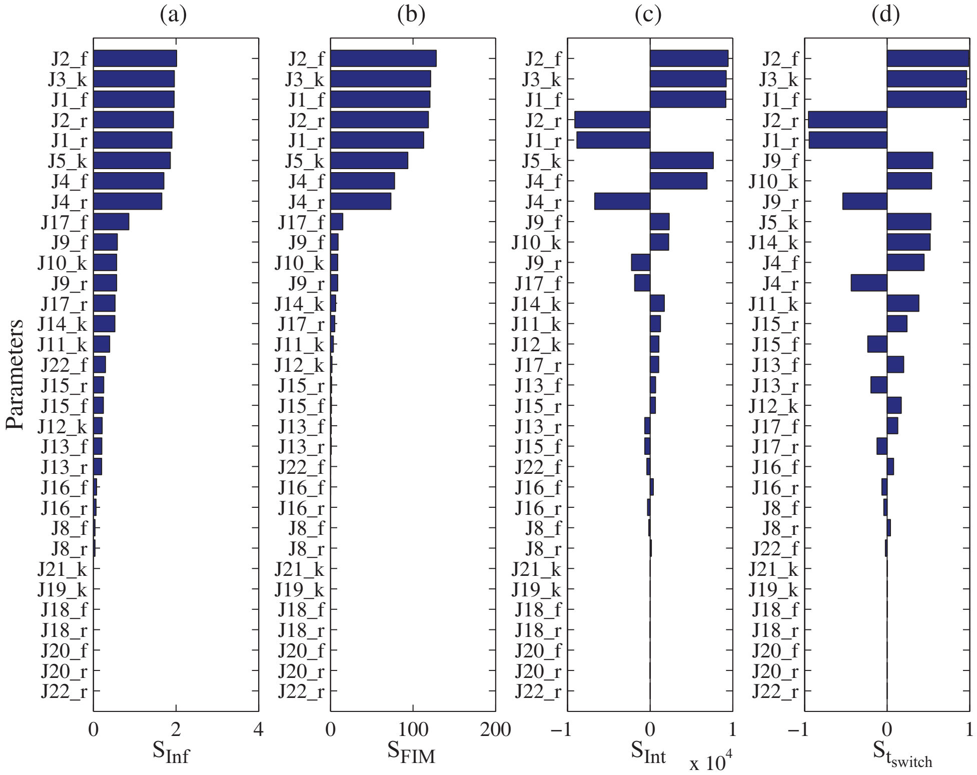

Supplementary Figure S3 - Local Parametric Sensitivity Analysis of caspase-3 activation under a constant FasL stimulus.

(a-c) The bar graphs represent consolidated sensitivity metrics of caspase-3: infinite norm, Fisher Information Matrix (FIM), time integrated sensitivity coefficients, respectively, with respect to the parameters indicated on the y-axis. (d) The PSA coefficients of caspase-3 magnitudes with respect to the same parameters at switching time (t=6060s). The parameter numbers refer to the reactions as shown in Figure 6, where the subscripts *f* and *r* denote forward and backward rate constants for reversible reactions and *k* denotes the rate constants for irreversible reactions.

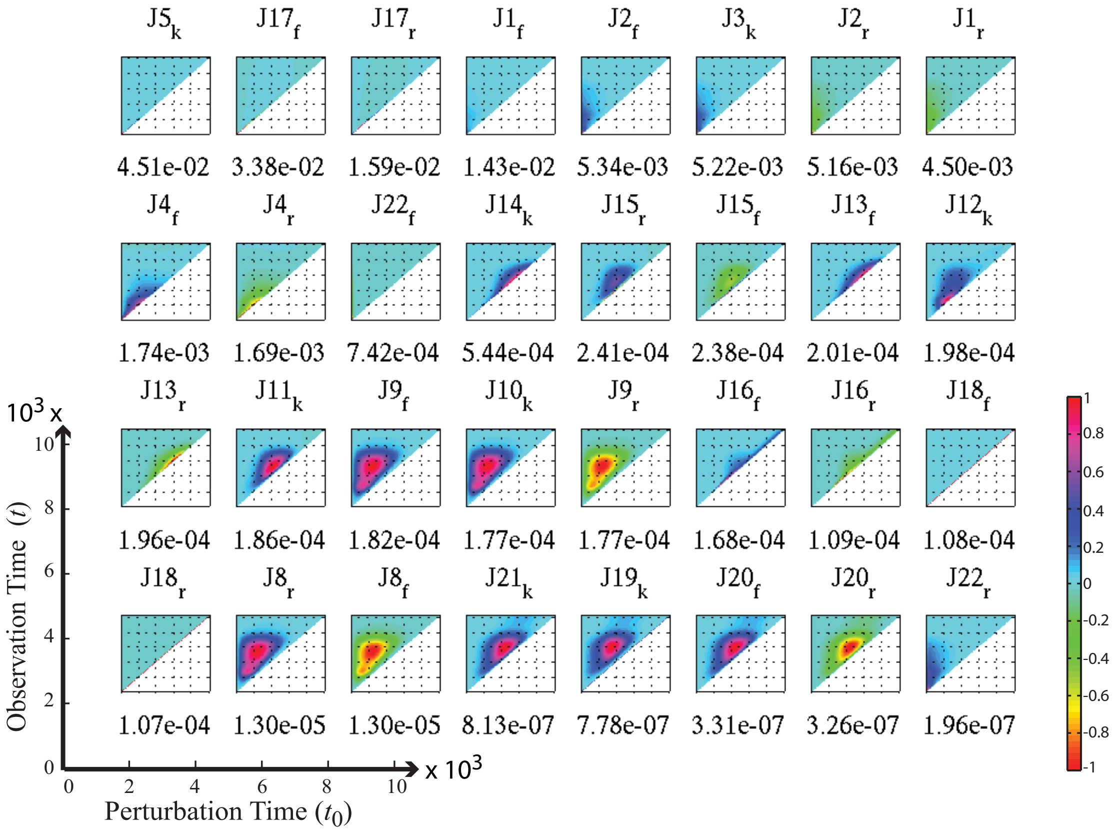

Supplementary Figure S4 - Impulse Parametric Sensitivity Analysis (iPSA) of caspase-3 activation by a constant FasL (2nM) stimulus

Each heatmap illustrates the iPSA coefficient of active caspase-3 level with respect to perturbations on one parameter in the network, indicated in the title. The x-axis gives the time at which impulse perturbation is applied to the parameter, while the y-axis indicates the observation time of caspase-3(t). For comparison purpose, each plot is scaled to have values between -1 and +1 by the scaling factor reported in the abscissa. The plots are arranged in the decreasing order of their scaling factors (left to right, top to bottom). The heatmap mainly suggests that: (a) the early activation of caspase-3 is due to the type-I pathway, and (b) the procaspase-3 cleaving by type –II pathway is active during the switching. Therefore, the iPSA suggests a type-II dependent activation of caspase-3.

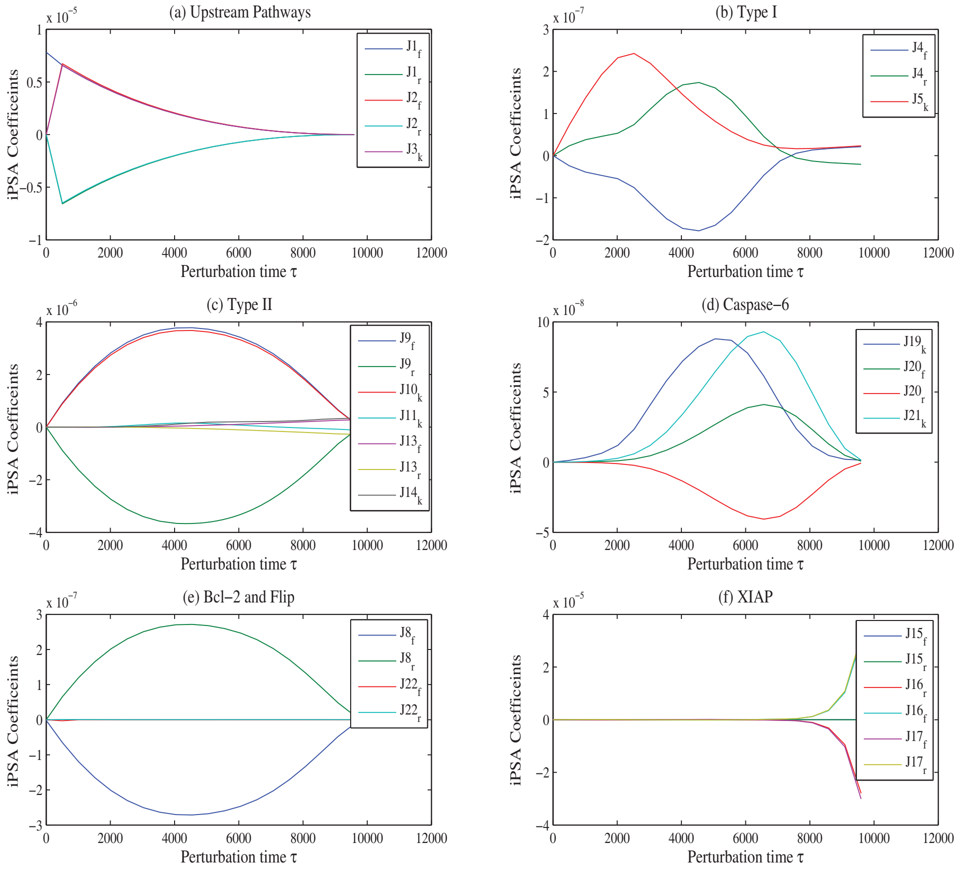

Supplementary Figure S5 - Impulse Parametric Sensitivity Analysis (iPSA) of caspase-3 at *t* = 10,000 seconds by a constant FasL (2nM) stimulus

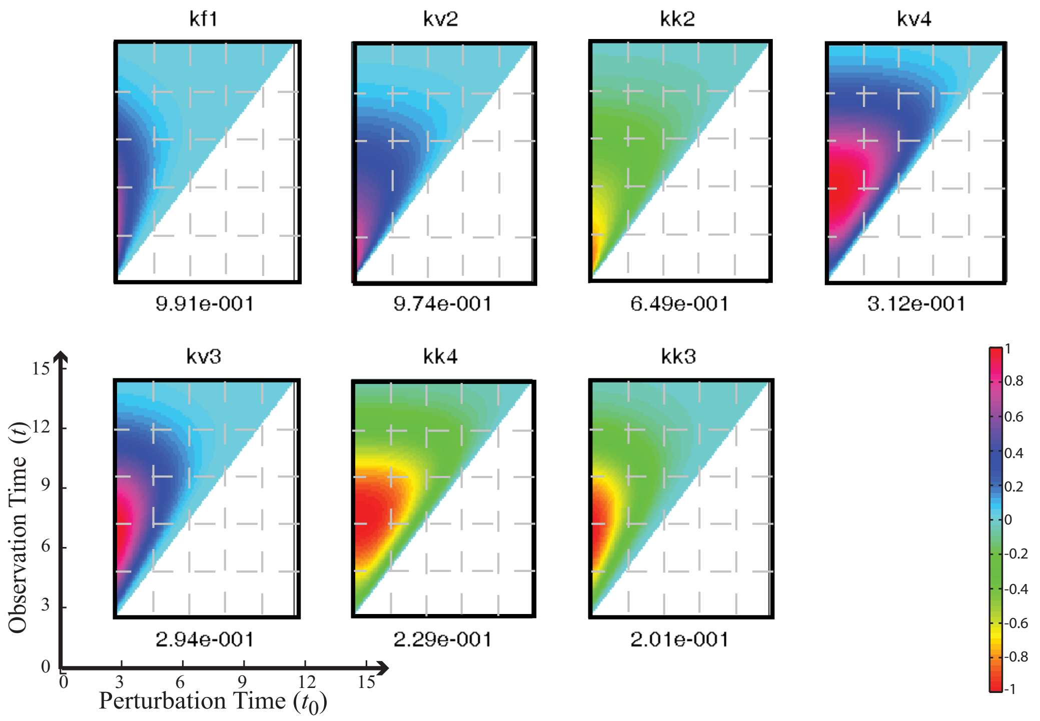

Supplementary Figure S6 - Local Parametric Sensitivity Analysis (PSA) of x_6 activation in response to x_1 stimulus

Each heatmap illustrates the local PSA coefficient of active x_6 level with respect to perturbations on one parameter in the network, indicated in the title. The x-axis gives the time at which perturbation is applied to the parameter, while the y-axis indicates the observation time of $x_6(t)$. For comparison purpose, each plot is scaled to have values between -1 and +1 by the scaling factor reported in the abscissa. The plots are arranged in the decreasing order of their scaling factors (left to right, top to bottom).

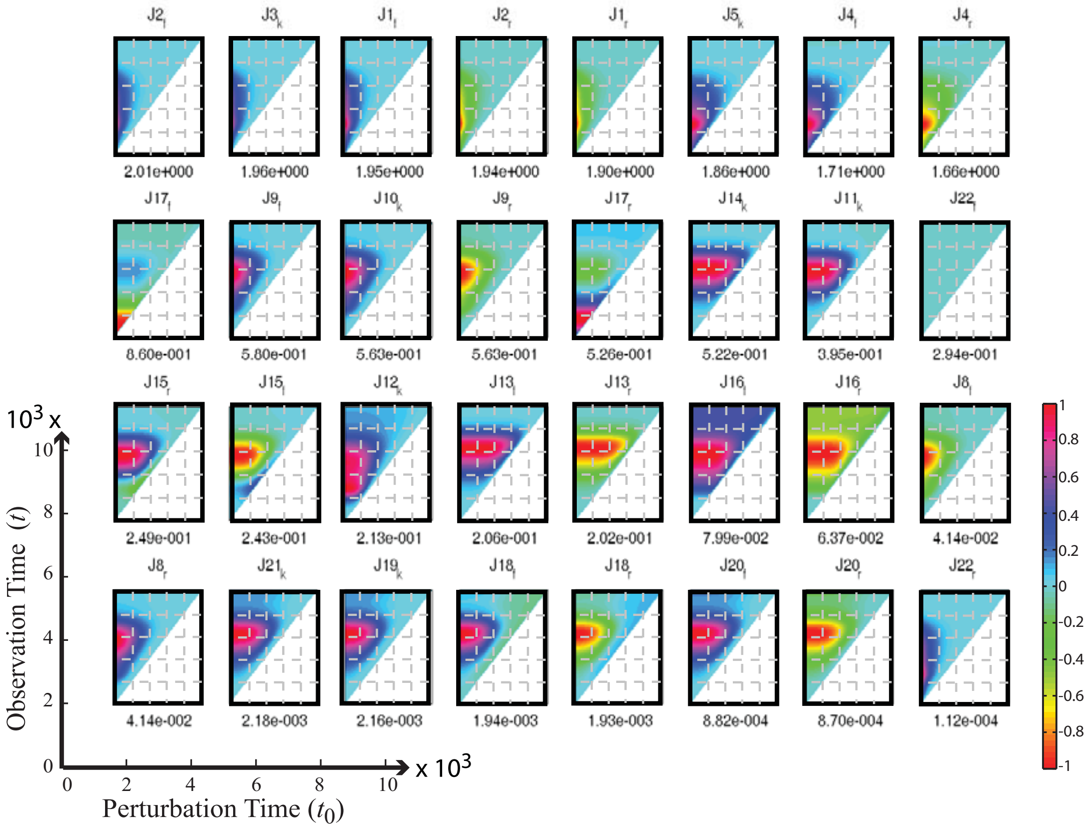

Supplementary Figure S7 - Local Parametric Sensitivity Analysis (PSA) of caspase-3 activation by a constant FasL (2nM) stimulus

Each heatmap illustrates the local PSA coefficient of active caspase-3 level with respect to perturbations on one parameter in the network, indicated in the title. The x-axis gives the time at which impulse perturbation is applied to the parameter, while the y-axis indicates the observation time of caspase-3(t). For comparison purpose, each plot is scaled to have values between -1 and +1 by the scaling factor reported in the abscissa. The plots are arranged in the decreasing order of their scaling factors (left to right, top to bottom).

Supplementary Table S1 - Simple Network Model

**Initial Conditions**			**Rate Constants**	
x_1	1		kf1	0.06
x_2	0		kv2	1
x_3	1		kk2	2
x_4	1		kv3	1
x_5	0		kk3	2
x_6	0		kv4	1
			kk4	2

**Reaction Number**	**Rate Equation**
r_1	kf1*x_1
r_2	kv2*x_2*x_4/(kk2+x_4)
r_3	kv3*x_2*x_3/(kk3+x_3)
r_4	kv4*x_5*x_4/(kk4+x_4)

**Differential Equations**	
dx_1/dt	$-r_1$
dx_2/dt	$+r_1$
dx_3/dt	$-r_3$
dx_4/dt	$-r_2 - r_4$
dx_5/dt	$+r_3$
dx_6/dt	$+r_2+r_4$

Supplementary Table S2 - Fas-induced apoptosis in human Jurkat cancer T-cell lines

Initial Conditions	nM	Rate Constants	
'Fas'	1.67E+01	'J1_f'	1.01E-02
'ProCaspase 8'	3.33E+01	'J1_r'	8.96E-01
'DISC'	0.00E+00	'J2_f'	8.89E-03
'DISC:Casp8'	0.00E+00	'J2_r'	7.85E-01
'Caspase-8'	0.00E+00	'J3_k'	2.94E-02
'ProCaspase-3'	2.00E+02	'J4_f'	4.38E-04
'Casp-8:Casp-3'	0.00E+00	'J4_r'	7.88E-01
'Caspase-3'	0.00E+00	'J5_k'	2.25E-02
'Bcl-2'	7.50E+01	'J8_f'	6.62E-02
'Bcl-2:Mito'	0.00E+00	'J8_r'	5.26E-03
'Mitochondria'	8.31E+01	'J9_f'	1.00E-05
'Caspase-8:Mitochondria'	0.00E+00	'J9_r'	7.67E-01
'Activated Mitochondria'	0.00E+00	'J11_k'	1.68E-02
'Apoptosome'	1.66E+01	'J12_k'	1.82E-03
'Activated Apoptosome'	0.00E+00	'J13_f'	8.44E-03
'Act. Apop.:Caspase-3'	0.00E+00	'J13_r'	5.91E-01
'SMAC'	1.00E+02	'J14_k'	1.25E-02
'Activated Smac'	0.00E+00	'J15_f'	9.03E-02
'XIAP'	3.00E+01	'J15_r'	1.02E-02
'Apoptosome:XIAP'	0.00E+00	'J16_r'	1.32E-03
'Activated Smac:XIAP'	0.00E+00	'J18_f'	1.08E-05
'Caspase-3:XIAP'	0.00E+00	'J18_r'	5.37E-01
'Caspase-3:Caspase-6'	0.00E+00	'J19_k'	1.97E-03
'Caspase-6'	0.00E+00	'J20_f'	2.68E-02
'Caspase-6:Caspase-8'	0.00E+00	'J20_r'	4.67E-01
'ProCaspase-6'	1.00E+01	'J21_k'	5.31E-03
'FLIP'	8.00E+00	'J22_f'	7.99E-02
'DISC:Flip'	0.00E+00	'J22_r'	1.00E-06
'FasLigand'	2.00E+00	'J16_f'	7.00E-03
		'J17_f'	2.50E-03
		'J17_r'	2.40E-03
		'J10_k'	2.25E-02

Rate Equations
r1_f = J1_f*FasL*FADD;
r1_r = J1_r*DISC;
r2_f = J2_f*Casp8*DISC;
r2_r = J2_r*Casp8_DISC;

r3_k = J3_k*Casp8_DISC;	
r4_f = J4_f*Casp8_act*Casp3;	
r4_r = J4_r*Casp3_Casp8_act;	
r5_k = J5_k*Casp3_Casp8_act;	
r8_f = J8_f*Bcl2*Mit;	
r8_r = J8_r*Bcl2_Mit;	
r9_f = J9_f*Casp8_act*Mit;	
r9_r = J9_r*Mit_Casp8_act;	
r10_k = J10_k*Mit_Casp8_act;	
r11_k = J11_k*Apopt*Mit_act;	
r12_k = J12_k*Smac*Mit_act;	
r13_f = J13_f*Apopt_act*Casp3;	
r13_r = J13_r*Casp3_Apopt_act;	
r14_k = J14_k*Casp3_Apopt_act;	
r15_f = J15_f*Apopt*XIAP;	
r15_r = J15_r*XIAP_Apopt;	
r16_f = J16_f*XIAP*Smac_act;	
r16_r = J16_r*XIAP_Smac;	
r17_f = J17_f*XIAP*Casp3_act;	
r17_r = J17_r*XIAP_Casp3_act;	
r18_f = J18_f*Casp3_act*Casp6;	
r18_r = J18_r*Casp6_Casp3_act;	
r19_k = J19_k*Casp6_Casp3_act;	
r20_f = J20_f*Casp8*Casp6_act;	
r20_r = J20_r*Casp8_Casp6_act;	
r21_k = J21_k*Casp8_Casp6_act;	
r22_f = J22_f*DISC*Flip;	
r22_r = J22_r*Flip_DISC;	
**Differential Equations**	
d(Fas)/dt	- r1_f + r1_r; % dFADD
d(ProCaspase 8)/dt	- r2_f + r2_r - r20_f + r20_r; % dCasp8
d(DISC)/dt	+r1_f - r1_r - r2_f + r2_r + r3_k - r22_f + r22_r;% dDISC
d(DISC:Casp8)/dt	+r2_f - r2_r - r3_k; % dCasp8_DISC
d(Caspase-8)/dt	+r3_k - r4_f + r4_r + r5_k - r9_f + r9_r + r10_k + r21_k ; % dCasp8_act
d(ProCaspase-3)/dt	-r4_f + r4_r - r13_f + r13_r ; % dCasp3
d(Casp-8:Casp-3)/dt	+r4_f - r4_r - r5_k ; % dCasp3_Casp8_act
d(Caspase-3)/dt	+r5_k + r14_k - r17_f + r17_r - r18_f + r18_r + r19_k ; % dCasp3_act
d(Bcl-2)/dt	-r8_f + r8_r ; % dBcl2
d(Bcl-2:Mito)/dt	+r8_f - r8_r ; % dBcl2_Mit
d(Mitochondria)/dt	-r8_f + r8_r - r9_f + r9_r ; % dMit

d(Caspase-8:Mitochondria)/dt	+r9_f - r9_r - r10_k ; % dMit_Casp8_act
d(Activated Mitochondria)/dt	+r10_k ; % dMit_act
d(Apoptosome)/dt	-r11_k - r15_f + r15_r ; % dApopt
d(Activated Apoptosome)/dt	+r11_k - r13_f + r13_r + r14_k ; % dApopt_act
d(Act. Apop.:Caspase-3)/dt	+r13_f - r13_r - r14_k ; % Casp3_Apopt_act
d(SMAC)/dt	-r12_k ; % dSmac
d(Activated Smac)/dt	+r12_k - r16_f + r16_r ; % dSmac_act
d(XIAP)/dt	-r15_f + r15_r - r16_f + r16_r - r17_f + r17_r ; % dydtIAP
d(Apoptosome:XIAP)/dt	+r15_f - r15_r ; % dydtIAP_Apopt
d(Activated Smac:XIAP)/dt	+r16_f - r16_r ; % dydtIAP_Smac
d(Caspase-3:XIAP)/dt	+r17_f - r17_r ; % dydtIAP_Casp3_act
d(Caspase-3:Caspase-6)/dt	+r18_f - r18_r - r19_k; % dCasp6_Casp3_act
d(Caspase-6)/dt	+r19_k - r20_f + r20_r + r21_k; % dCasp6_act
d(Caspase-6:Caspase-8)/dt	+r20_f - r20_r - r21_k; % dCasp8_Casp6_act
d(ProCaspase-6)/dt	-r18_f + r18_r; % dCasp6
d(FLIP)/dt	-r22_f + r22_r; % dFlip
d(DISC:Flip)/dt	+r22_f - r22_r; % dFlip_DISC

Detailed initial conditions, rate constants, rate equations and differential equations of Fas-induced apoptotic cell death model adopted from [1]. The model has 28 states and 32 rate constants.

Reference:

1. Hua F, Hautaniemi S, Yokoo R, Lauffenburger DA: **Integrated mechanistic and data-driven modelling for multivariate analysis of signalling pathways.** *J R Soc Interface* 2006, **3:**515--526.
